# Effects of fertilizer application schemes and soil environmental factors on nitrous oxide emission fluxes in a rice-wheat cropping system, east China

**DOI:** 10.1371/journal.pone.0202016

**Published:** 2018-08-14

**Authors:** Awais Shakoor, Yunlian Xu, Qiang Wang, Ningyi Chen, Fei He, Huaifeng Zuo, Hanxun Yin, Xiaoyuan Yan, Youhua Ma, Shuyun Yang

**Affiliations:** 1 School of Resources and Environment, Anhui Agricultural University, Hefei, China; 2 The Institute of Soil Science, Chinese Academy of Sciences, Nanjing, China; Tennessee State University, UNITED STATES

## Abstract

Nitrous oxide (N_2_O) is a potent greenhouse gas (GHG) with agricultural soils representing its largest anthropogenic source. However, the mechanisms involved in the N_2_O emission and factors affecting N_2_O emission fluxes in response to various nitrogenous fertilizer applications remain uncertain. We conducted a four-year (2012–2015) field experiment to assess how fertilization scheme impacts N_2_O emissions from a rice-wheat cropping system in eastern China. The fertilizer treatments included Control (CK), Conventional fertilizer (CF), CF with shallow-irrigation (CF+SI), CF with deep-irrigation system (CF+DI), Optimized fertilizer (OF), OF with Urease inhibitor (OF+UI), OF with conservation tillage (OF+CT) and Slow-release fertilizer (SRF). N_2_O emissions were measured by a closed static chamber method. N_2_O emission fluxes ranged from 0.61 μg m^-2^ h^-1^ to 1707 μg m^-2^ h^-1^, indicating a significant impact of nitrogen fertilizer and cropping type on N_2_O emissions. The highest crop yields for wheat (3515–3667 kg ha^-1^) and rice (8633–8990 kg ha^-1^) were observed under the SRF and OF+UI treatments with significant reduction in N_2_O emissions by 16.94–21.20% and 5.55–7.93%, respectively. Our findings suggest that the SRF and OF+UI treatments can be effective in achieving maximum crop yield and lowering N_2_O emissions for the rice-wheat cropping system in eastern China.

## Introduction

Following carbon dioxide (CO_2_) and methane, nitrous oxide (N_2_O) is the 3^rd^ most important anthropogenic greenhouse gas (GHG) and contributes up to 6–10% in global warming [1]. N_2_O is a long-lived GHG with a lifespan of over 114 years in the atmosphere [2]. N_2_O has 298 times global warming potential (GWP) as compared to CO_2_ and it also has a great potential for O_3_ destruction [2–4]. From 1750 to 2011, the atmospheric N_2_O concentration has increased from 271 parts per billion (ppb) to 324.2 ppb [5]. Agricultural soils contributed about 60% of the anthropogenic N_2_O emissions, and this was mostly due to increased chemical fertilizer application [3,6]. In addition, humankind’s increased fossil fuel combustion and continuous use of nitrogen based fertilizer in agriculture affects the global nitrogen biogeochemical cycle [7,8]. Due to increases in food demands, emissions of N_2_O from agricultural soil are expected to rise to 6–7 Tg N/year by 2030 [9,10]. In most agricultural soils, N_2_O is formed biologically via nitrification and denitrification, and these microbial processes are strongly affected by natural conditions and agricultural management practices [11]. Greenhouse gas emissions intensity (GHGI) is defined as GWP (global warming potential) per unit crop yield. It is suitable for determining N_2_O emission factors and for checking the impact of different kinds of agricultural practices on the environmental ecosystem and global climate change [12,13].

Worldwide, China ranks first in agricultural output, and is critically important for meeting global food demand [14,15]. To increase crop yield, several new agricultural management practices such as improved irrigation, fertilization and crop rotation systems are used for intensive agricultural production in China[11].The annual summer rice/winter wheat crop rotation system is an important double cropping system widely used in eastern part of China [9,13]. Over the past few years, the fertilizer application rate has been increased to maximize crop production, but this has had adverse effects on the terrestrial environment as well as the atmosphere. Agricultural practices, especially the application of nitrogenous fertilizers (N-Fertilizer), have a major influence on soil N_2_O emissions [2,11,16]. The excessive use of nitrogenous fertilizers are pervasive and have resulted in many environmental problems, including soil acidification, pollution of water, soil salination and emission of GHGs [11,17]. The annual application rate of nitrogenous fertilizer in vegetable fields is around 1000 to 1500 kg N per hectare (ha) [2,18], but some agricultural fields in China use more than 2800 kg N per ha per year [19]. As a result, the overuse of nitrogenous fertilizer with low N use efficiency in agricultural fields has resulted in multiple environmental and agricultural issues [20,21].The rice-wheat crop rotation cycle is a very important agricultural practice for increasing land use efficiency and crop yield in east China. In a rice-wheat cropping system, increases in the application of nitrogenous fertilizer could lead to the emergence of N_2_O emission peaks (in the range of 0~225kg N ha^-1^). Previous studies have reported that fertilization enhances N_2_O emission from agricultural soils [9,11]. In general, there is a strong increase in the emission of N_2_O associating with nitrogen application rates in agricultural soils [22,23]. A researcher reported a non-linear exponentially elevating N_2_O emissions response to nitrogen application rates from a soybean-corn rotation [24] with N_2_O emissions not significantly decreasing with reductions in nitrogen fertilizer application rates in a wheat-maize rotation cycle [25]. Comparatively, there were very few studies that measured N_2_O emission fluxes from rice-wheat cropping systems, especially in Chaohu Basin, China [26].However, the mechanisms involved in the N_2_O emission under various agricultural practices, their flux in response to various nitrogenous fertilizer (N-fertilizer) applications and factors affecting N_2_O emission fluxes remain unclear.

In this study, we investigated four-year N_2_O emissions from soil and their responses to different N fertilizer application schemes in a rice-wheat cropping system in east China. Additionally, we also studied the impact of environmental factors (soil temperature, precipitation, air temperature, soil conductivity and water-filled pore space (WFPS)) on N_2_O emission fluxes and crop yield. GWP and Greenhouse gas emission intensity (GHGI) under different fertilization treatments were also measured. The main objectives and aims to run this research experiment include: 1) To determine the level of GWP of GHGs emissions around the research station and the community where they were sited, 2) To illustrate the level and extent of environmental hazards and disasters caused by GHGs emissions in the catchment area of the research site, and 3) Determination the anthropogenic sources that were involved in the GHGs emissions and climate change.This study was helpful to overcome the GWP of GHGs from rice-wheat cropping system in eastern China.

## Materials and methods

### Description of study site

This study was undertaken in a research facility center of Anhui Agricultural University, Hefei, China. The long-term monitoring point of this experiment is located in Xi Song Village, Chaohu, Anhui province, China. The specific location is 117° 40' 48 "east longitude and 31° 39 '57" north latitude, and is 17 m above sea level. The climate in this area is characterized by a subtropical humid monsoon climate. The annual average temperature is 15.7°C and the average annual rainfall is 1039.4 mm. From 1986 to 2005, the mean seasonal temperature was 16.29°C, which was similar to our findings [27]. A rice-wheat crop rotation pattern is typically practiced in this area. A rice-wheat rotation cycle was undertaken in this experimental farm from 2008 prior to initiating this experiment in 2012. Soil Electrical conductivity (EC) was also measured by using EC meter. The soil type at the monitoring site is clay loam (sand 30%, silt 35%, and clay 35%) that having maximum water holding capacity. The physical and chemical properties of soil (0–20 cm) were: pH (H_2_O) 6.18; organic matter 23.64 g kg^-1^; total nitrogen 1.30 g kg^-1^, respectively. During the whole experimental period, no animals were used or harmed.

### Experimental design and field management

The 2012–2015 of rice-wheat rotation field experiment was conducted with a randomized complete block design (RCBD). This experiment was started on 25 May 2012 and completed on 20 May 2015. Eight different fertilization treatments were used over the course of the experiment (S1 Table). Three replications of each fertilizer treatment were performed with an experimental plot area of 30 m^2^. The names of all fertilizer treatments were: Control (CK), Conventional fertilizer (CF), CF with shallow irrigation (CF+SI), CF with deep irrigation (CF+DI) system, Optimized fertilizer (OF), OF with Urease inhibitor (OF+UI), OF with conservation tillage (OF+CT) and slow release fertilizer (SRF). Urea, single super phosphate (SSP) and Potassium chloride (KCl) was used as a source of nitrogen (N), phosphorus (P) and potassium (K), respectively. The amount of irrigation water for DI and SI treatments were 822.7 mm and 655.2 mm, respectively. UI hydroquinone, also known as hydroquinone with molecular formula C_6_H_4_ (OH) _2_ or ‎C_6_H_6_O_2_, was used with urea during the experiment and was purchased from Wuxi City Pharmaceutical production Co., Ltd. UI hydroquinone was applied at the rate of 112.09 kg ha^-1^ of soil. Polymer coated fertilizer (PCF) was used for all SRF experimental treatments (Anhui Di Yuan Biotechnology Co. Ltd). Zero/no-tillage practice was used as a conservation tillage practice.

Every year, the rice crop was planted in May and harvested in early October, while the wheat crop was sown in mid-October and harvested at the end of May. Rice and wheat cultivars named “Longping0293” and “Ningmai16” were bought from Wuhan Comega Seed Co., Ltd. These are both high yielding cultivars, and are mainly cultivated in Anhui province. Rice plants were transplanted to the main field at a density of 20 hills per m^2^ on May 25/26 and harvested on October 10/11 for the entire experimental period. The application rate of nitrogen fertilizer was 225 kg ha^−1^, and was applied at a ratio of 5:3:2 (w/w/w) at the basal, tillering and heading stages. Basal fertilizer was applied to the rice crop after transplanting into the main field, and the topdressing was applied at the tillering and heading stages. Whole Phosphorous (P_2_O_5_) fertilizer and 45% potassium (K_2_O) fertilizer was applied at the basal stage, but the remaining K_2_O fertilizer was applied at the heading stage in the form KCl. For the wheat crop, basal fertilizer was applied at the time of sowing and further fertilizer was applied at the tillering and panicle stages. The complete fertilizer application plan used during the experiment is shown in S1 Table.

Fertilization has an important impact on crop yield and its composition, as well as greenhouse gas emissions. In order to analyze the specific effect of different fertilizer treatments on crop yield, the crop yield was measured in the plot. At the same time, some plant samples were used to calculate the number of grains per spike and the 1000-grain weight. Over the entire experimental period, the application rates of N-fertilizer for each treatment were the same and ranged from 0 to 225 kg ha^-1^. WFPS was calculated based on the determined volumetric water content (VWC), soil bulk density of 1.17gcm^−3^ and soil particle density of 2.65gcm^−3^. Air temperature and precipitation were recorded at a nearby metrological station.

### Sample collection and N_2_O fluxes measurement

A static closed chamber was constructed with polyester material, and was used to measure the N_2_O fluxes [9,28]; the height of the static chamber was 1 m along with 0.5 m width and length. The base of the chamber was made of PVC material (0.5 m × 0.5 m × 0.15 m) that was installed to a depth of 10 cm in the soil. There were three manual static chambers used in each plot for sample collection. All chambers were wrapped with aluminum foil to control chamber air temperature and equipped with a circulating fan to ensure complete gas mixing throughout the sampling period. We collected three different gas samples (n = 3) using a 50-mL plastic syringe from each static chamber at six minutes time intervals after closing the chamber.

For the rice-wheat cropping seasons, N_2_O fluxes were calculated between 25 May to 10 October and 15 October to 20 May (2012–2015), respectively. N_2_O gas samples were collected between 8:00 and 11:00 am from the experimental field. The measurements were taken at intervals of 3, 5 or 7 days used to estimate seasonal N_2_O emission values. After collection, the gas samples were immediately taken from the field to the laboratory for analysis. The gas samples were analyzed for their N_2_O and CH_4_ contents using a gas chromatograph (Bruker 450-GC, USA) after 24 h sample collection. N_2_O was detected with the Ni63ECD detector and a 300°C detector temperature; the flow rate of nitrogen was 300 mL min^-1^. CH_4_ was analyzed on the FID channel with 300 detector temperature and helium gas was used to measure the CH_4_ emission flux. We measured CH_4_ fluxes only to calculate the GWP. GHG emission fluxes (N_2_O/CH_4_ flux) from farmland were determined by using the following equation.

F=p∙V/A∙dc/dt∙273/(273+T) (1)

Where: F is the rate of N_2_O flux (mg m^-2^h^-1^), p is the N_2_O density (N_2_O: 1.25 kg m^-3^) under standard conditions, V is the volume of the chamber (m^3^), A is the area of the chamber base (m^2^), V/A for the chamber height, dc/dt is the change rate of GHG concentration in the sampling chamber (mL m^-3^ h^-1^) and T is the mean temperature inside the chamber.

The contribution of GHG emissions to global warming is estimated in terms of CO_2_ equivalents based on the integrated global warming potential (GWP) [29]. The total equivalent CO_2_ for N_2_O and CH_4_ flux emissions were estimated by using following equation.

CO2‑eq=25RCH4+298RN2O (2)

Where CO_2-eq_ is the total emission of CO_2_ equivalent (kgCO_2-eq_ ha^-1^) per unit area during the growing season, and RCH4 and RN2O are the total amounts of CH_4_ and N_2_O emissions (kg ha^-1^), 25 and 298 refer to the respective multiples of GWP for N_2_O and CH_4_ flux emission over a given time horizon (typically 100 years).

In order to reflect the environmental and economic benefits of crops, the greenhouse gas emission intensity (GHGI) was proposed as a comprehensive index, which is the corresponding CO_2-eq_ of per unit crop yield [30].

GHGI=CO2‑eq/cropyieldperunitarea (3)

### Statistical analysis

All statistical analyses were performed using SPSS 17.0 (SPSS, Inc., USA) and EXCEL 2010 for Windows. Average fluxes and standard deviations of N_2_O were calculated based on data from triplicate plots. Differences in seasonal cumulative N_2_O emissions and rice-wheat crop yields as affected by nitrogen fertilizer were examined. Differences in seasonal N_2_O emissions and grain yields between treatments were analyzed with two-way analysis of variance (ANOVA) and least significant difference (LSD) test at a significance level of *P*<0.05. Finally, Origin 8.0 (Origin Lab Corporation, USA) was employed to construct the figures.

## Results

### Environmental factors

**Fig 1:**
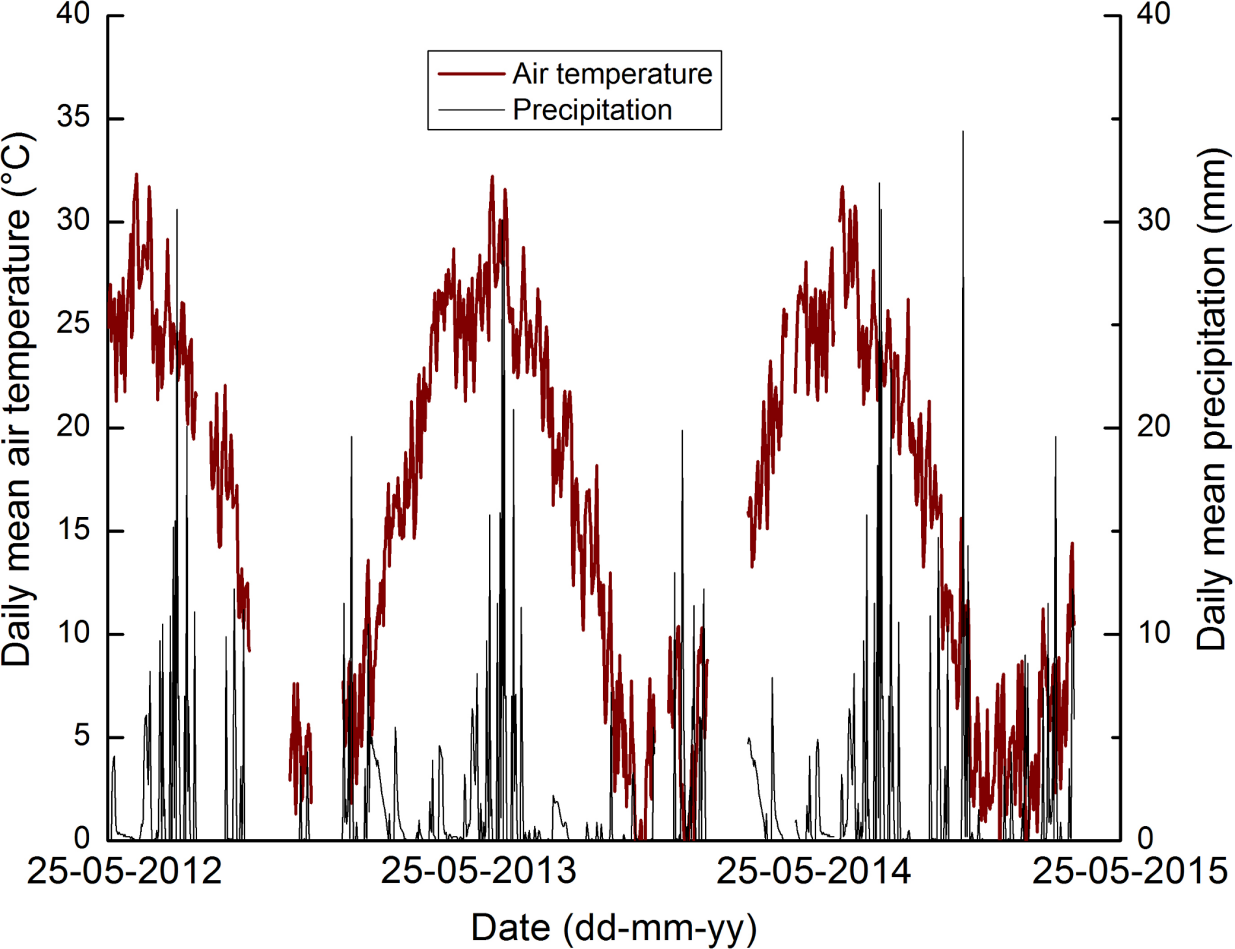
Distribution of daily precipitation (mm) and daily mean air temperature (°C) for the experimental period of 2012–2015 in Chaohu, China.

**Fig 2:**
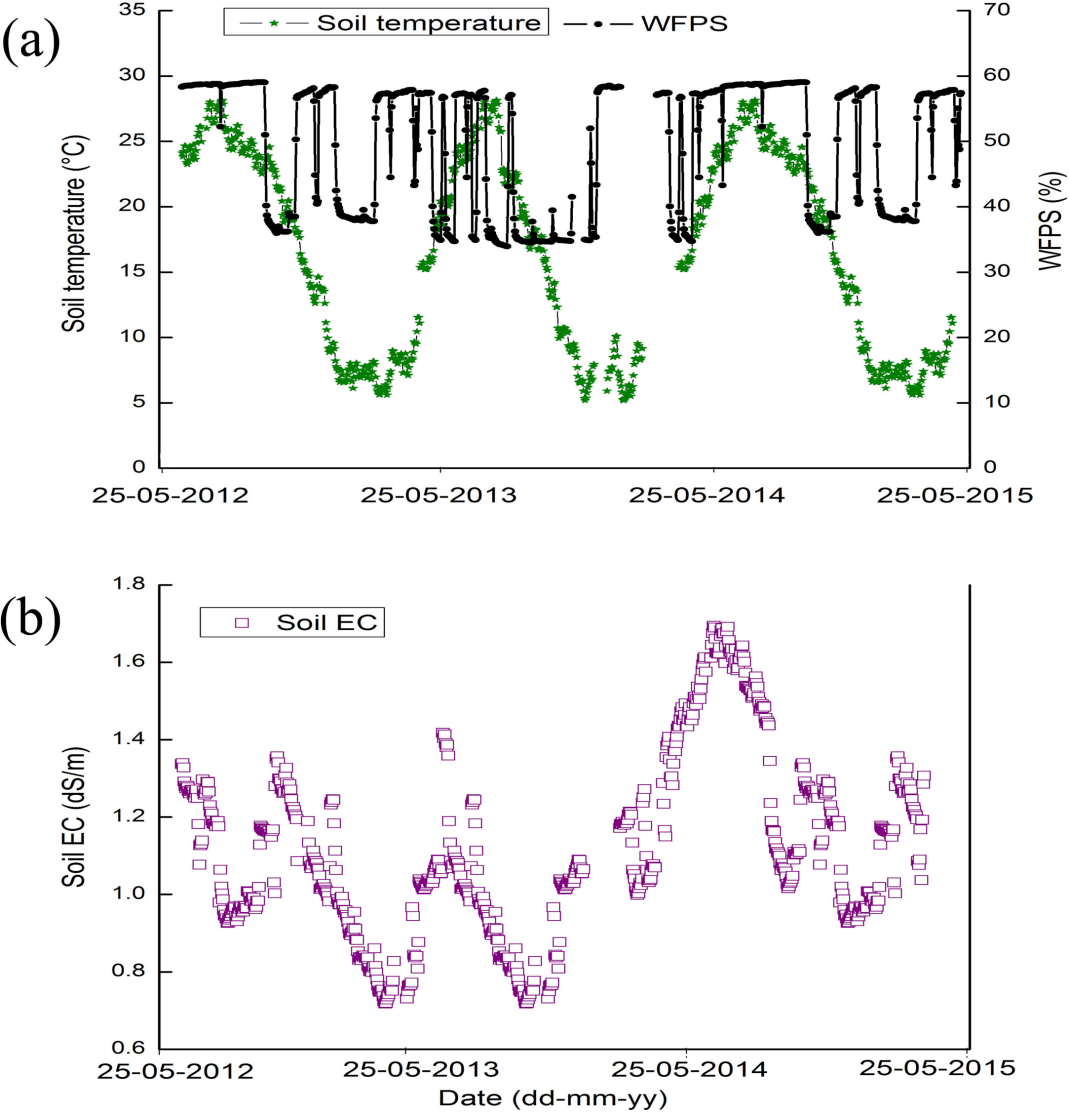
**Seasonal variation in (a) daily soil temperature°C (0–10 cm), and water-filled pore space (WFPS %), and (b) daily changes in soil electrical conductivity (EC, dS/m) in the rice-wheat cropping system from 2012–2015**.

During the 2012–2015 study period, the mean annual precipitation ranged between 931.7 and 1039.4 mm (Fig 1). Most of the precipitation occurred from July to November each year. Mean annual air temperature varied from 15.6°C to 15.7°C (Fig 1). WFPS contents ranged from 35.1% to 58.6% and average soil temperature varied from 7.1°C to 27.9°C (Fig 2A). During the 2013–14 and 2014–15 experimental period, the percentage of WFPS ranged from 34.9% to 59.2% and 38.7% to 58.6%, respectively; similarly, the soil temperature ranged from 7.1°C to 25.8°C and 7.1°C to 25.9°C, respectively (Fig 2A). The annual average soil electrical conductivity (EC) ranged from 1.0 to 1.1 dS m^-1^ during the experimental period (Fig 2B).

### Nitrous oxide fluxes

**Fig 3:**
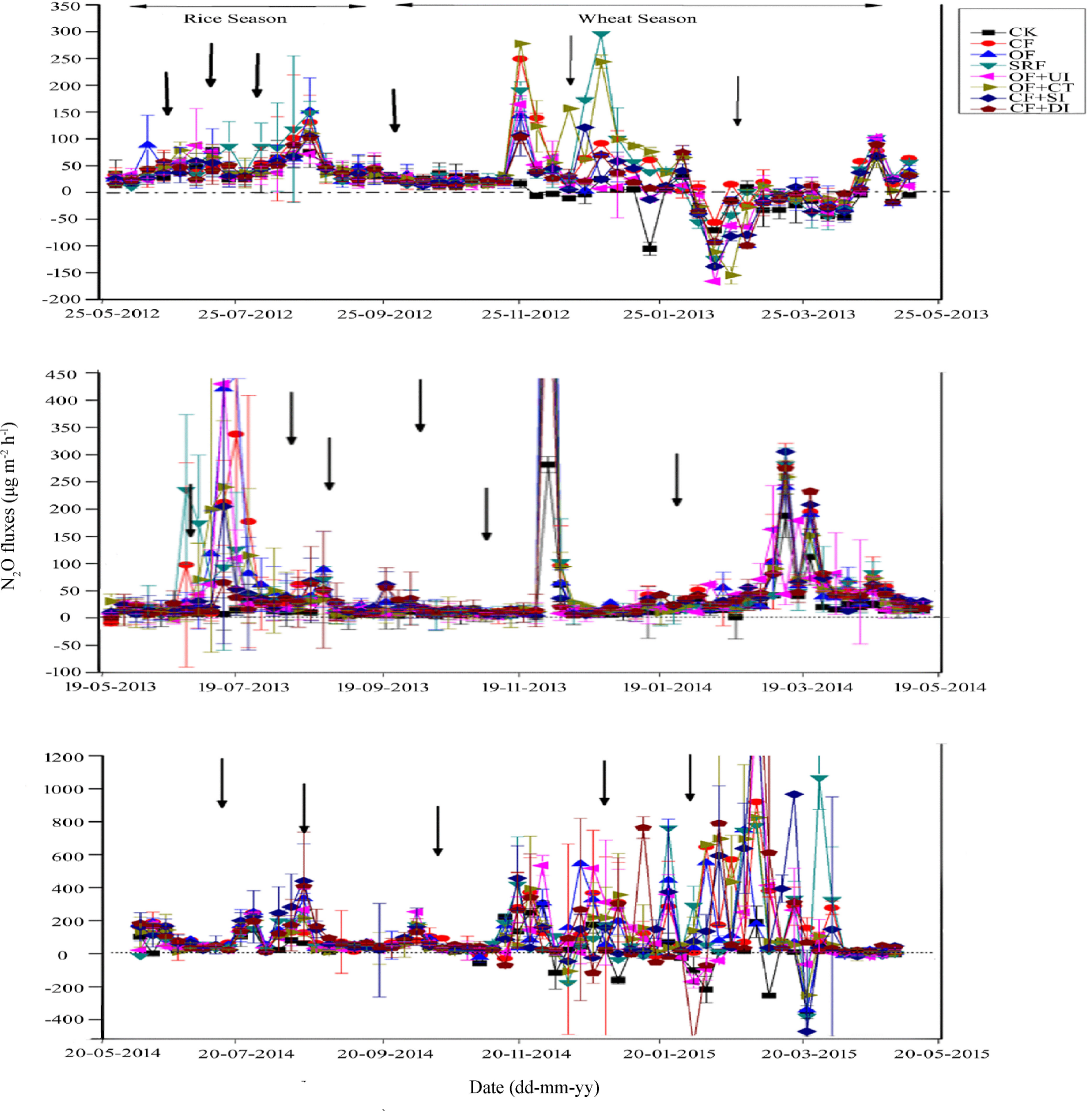
Seasonal variation of nitrous oxide (N_2_O) (μg m^-2^ h^-1^) emission fluxes from rice-wheat cropping systems in three annual cycles during the period of 2012–2015. The error bars show standard errors of the mean (n = 3) and arrows indicate fertilizer application times.

The fluxes of N_2_O emissions from rice-wheat cropping fields ranged between 0.61 μg m^-2^ h^-1^ to 1707.08 μg m^-2^ h^-1^ over the entire experiment (Fig 3). Negative N_2_O fluxes (range –0.5 μg m^-2^ h^-1^ to –378.55 μg m^-2^ h^-1^) were also observed mostly during the wheat cropping season (Fig 3). As shown in our results, the N_2_O emission peaks occurred from 0 to 7 days after fertilization in the rice-wheat cropping. Mostly peak fluxes were observed in wheat cropping seasons. Taking the OF treatment as an example, emission peaks occurred on the 2^nd^ and 6^th^ days after applying basal fertilizer and tillering stage fertilizer in wheat crop, respectively; for rice, peak emissions occurred on the 2^nd^, 5^th^ and 7^th^ day after application of basal fertilizer, tillering fertilizer and panicle fertilizer, respectively.

The patterns in the timing of N_2_O emission fluxes from different treatments to the rice-wheat cropping system were approximately the same. In the rice season, the greatest emission peaks were observed after the transplanting and tillering stage, while in the wheat season, most of the peaks were observed at the tillering, booting and grain filling stages. The mean N_2_O emission fluxes were 21.44 ± 1.4, 77.42 ± 6.2, 68.35 ± 5.5, 70.77 ± 6.0, 62.88 ± 7.1, 71.02 ± 6.2, 72.93 ± 7.0, 66.38 ± 5.8 μg m^-2^ h^-1^for CK, CF, OF, SRF, OF+UI, OF+CT, CF+SI and CF+DI, respectively. The distribution patterns of N_2_O emissions were different during different growth stages (tillering, booting and grain filling) in both cropping seasons. The vegetative growth stage (germination to panicle initiation) was the main stage of N_2_O emission in the rice-wheat cropping system. In this stage, the proportion of N_2_O emissions from rice and wheat was 57~69% and 76~81%, respectively.

The values of cumulative N_2_O emissions differed during the whole experimental period within the same treatments. During the wheat season, the cumulative N_2_O emissions for OF, SRF and OF+UI were 115.90 ± 12.9 mg m^-2^, 96.44 ± 5.3 mg m^-2^ and 79.73 ± 4.4 mg m^-2^,and in the rice season, cumulative N_2_O emissions were 92.23 ± 9.67 mg m^-2^, 71.99 ± 5.43 mg m^-2^ and 54.87 ± 4.33 mg m^-2^, respectively. The highest GWP of N_2_O emissions were 0.21 ± 0.02 kg ha^-1^ (OF) in wheat and 1.20 ± 0.02 kg ha^-1^ (OF) in rice season (Table 1).

**Table 1:** Cumulative N_2_O fluxes and estimated GWP (global warming potential) under different fertilization treatments in the rice-wheat cropping system.

	Treatment	N_2_O		Integrated greenhouse effect kg CO_2_ ha^-1^	GWP CO_2_-eqkg ha^-1^
		Total emission mg m^-2^	Greenhouse effect kg CO_2_ ha^-1^		
Wheat	CK	-33.73±2.43b	-100.51±12.1b	-33.95±2.98b	-0.03±0.01b
	CF	102.41±14.8a	305.18±19.9a	473.72±9.43a	0.26±0.03a
	OF	115.90±12.9a	345.39±15.7a	504.41±13.5a	0.21±0.02a
	SRF	96.44±5.3a	287.40±11.3a	379.22±7.65a	0.17±0.01a
	OF+UI	79.73±4.4a	237.61±9.5a	384.17±5.98a	0.15±0.01a
	OF+CT	99.3±3.45a	295.8±14.6a	395.98±12.87a	0.8±0.02a
	CF+SI	89.4±9.2b	277.18±7.41b	359.43±8.98b	0.16±0.01b
	CF+DI	97.43±16.3a	290.76±9.72a	390.98±14.65a	0.20±0.02a
Rice	CK	19.99±1.32b	59.57±3.89b	6122.07±22.98a	1.04±0.01a
	CF	74.55±5.76a	222.16±8.90a	8774.66±36.12a	1.17±0.03a
	OF	92.23±9.67a	274.85±11.3a	9102.35±43.32a	1.20±0.02a
	SRF	71.99±5.43a	214.53±9.43a	8177.03±28.65a	1.15±0.01a
	OF+UI	54.87±4.33a	163.51±5.87a	7906.01±22.36a	1.00±0.01a
	OF+CT	86.98±5.98a	265.89±7.43a	7995.78±27.98a	1.18±0.02a
	CF+SI	62.67±5.98a	203.67±9.98a	8069.61±32.65a	1.14±0.01a
	CF+DI	85.32±9.76a	240.98±12.87a	8976.54±28.98a	1.15±0.01a

Lowercase letters indicate significant differences between treatments (*P*<0.05), and while capital letters indicate significant differences between treatments (*P*<0.01); ± show the standard errors (n = 3) of the replications.

Overall, the CK treatment showed the lowest peaks of seasonal N_2_O emissions in the rice-wheat cropping system. The CF treatment had the highest emissions during the wheat cropping season, whereas the OF treatment had the highest emissions during the rice cropping season. Compared with the CF treatment, the annual N_2_O emissions of the OF, SRF, OF+UI, CF+SI and CF+DI treatments showed highly significant reductions of 12.87%, 16.94%, 21.20%, 18.05% and 22.15% during the wheat cropping season, respectively (*P*<0.05, Table 1). In the rice cropping season, the annual N_2_O emissions of the SRF treatment were significantly reduced by 5.55%, and the reduction of OF+UI was extremely significant at 7.93%. The greenhouse gas emission reductions of SRF and OF+UI were the best among all treatments.

### Crop yield and equivalent CO_2_ emissions (CO_2-eq_) under different fertilization treatments

Application of higher amounts of nitrogen fertilizer enhanced crop yield.Relative to CK, the yield of wheat was increased by more than 120% for all treatments; similarly, the rice yield was increased by more than 40%, while the grain numbers and 1000-grain weights were also significantly increased. During the entire experimental period, the crop yields of CF+SI and CF+DI were increased by up to 12.11% (wheat), 5.51% (rice) and 11.32% (wheat), 2.98% (rice), respectively. The SRF treatment also had significantly increased crop yield over the experiment. The OF+UI treatment resulted in significant yield increases of up to 15.59% (wheat), 7.32% (rice) for the rice-wheat crop rotation cycle. OF+UI had the highest crop yield relative to other treatments. It can be seen that increasing the proportion of P and K fertilizers, optimizing fertilizer application and combining fertilizer with urease inhibitor can increase the grain number per spike and 1000-grain weight of the crop, thereby increasing the yield (Table 2).

**Table 2:** Total crop yield kg ha^-1^ for the rice-wheat cropping system.

	Treatment	Grains per spike	1000 grain weight g	Yield kg ha^-1^	Yield%
Wheat	CK	26±2	31.7±1.3	1438.9±68.3cC	-
	CF	31±2	42.2±1.3	3172.2±53.0bB	
	OF	33±2	46.3±0.7	3538.9±192.8aAB	11.56
	SRF	32±4	44.1±1.1	3514.6±51.8aAB	10.79
	OF+UI	34±2	46.2±1.2	3666.7±48.1aA	15.59
	OF+CT	31±2	38.3±0.9	3031.7±52.8aAB	9.98
	CF+SI	32±3	40.1±1.9	3287.1±81.8aA	12.11
	CF+DI	31.9±2	41.2±1.2	3266.5±51.3bB	11.32
Rice	CK	151±8	20.3±0.9	5966.7±135.6cB	-
	CFT	220±6	25.5±0.9	8376.7±189.6bA	
	OPT	239±7	26.1±1.3	8891.1±111.1aA	6.14
	SRF	229±11	25.9±1.6	8633.3±155.0abA	3.06
	OF+UI	244±7	26.7±0.6	8990.0±140.1aA	7.32
	OF+CT	239±7	25.9±1.6	8697.9±121.3aA	3.87
	CF+SI	204±9	22.1±1.7	7287.2±103.9bA	5.51
	CF+DI	230±8	26.1±1.3	8981.1±141.1aA	2.98

Lowercase letters indicate significant differences between treatments (*P*<0.05), and while capital letters indicate significant differences between treatments (*P*<0.01); ± show the standard errors (n = 3) of the replications.

Interestingly, there were significantly differences in CO_2-eq_ emissions among the treatments (*P*<0.05, Table 3). Over the experimental period, the total CO_2-eq_ emissions ranged from 5884 ± 351 CO_2_-eq kg ha^-1^ to 10864 ± 516 CO_2_-eq kg ha^-1^ and 341 ± 24 CO_2_-eq kg ha^-1^ to 749 ± 14 CO_2_-eq kg ha^-1^ for rice and wheat, respectively. During the rice season, the highest CO_2-eq_ emission was observed in the CF+DI treatment and the lowest CO_2eq_ emission was found in the CK treatment; whereas, during the wheat season, the highest and lowest CO_2-eq_ emission concentrations were observed in CF and CK, respectively (Table 3). In all treatments, the emissions of CO_2-eq_ were higher during the rice season as compared to the wheat season, which could be due to the reducing environment of paddy fields, which favors methanogenesis. The emissions of CO_2-eq_ varied considerably between rice growing seasons.

**Table 3:** Greenhouse gas emission intensity (GHGI) under different fertilization treatments in the rice-wheat cropping system.

	Treatment	TotalCO_2_-eq kg ha^-1^	Yield kg ha^-1^	GHGI kg kg^-1^	Reduction %
Wheat	CK	341.02±24.48dC	1438.9±68.3cC	0.24±0.03aA	——
	CF	749.25±14.09aA	3172.2±53.0bB	0.23±0.01aA	
	OF	650.61±0.45bB	3538.9±192.8aAB	0.19±0.01bAB	17.39
	SRF	603.77±10.09cB	3514.6±51.8aAB	0.17±0.01bB	26.09
	OF+UI	592.56±0.59aC	3666.7±48.1aA	0.16±0.01bB	30.43
	OF+CT	690.61±3.45aB	3031.7±52.8aAB	0.22±0.01bAB	20.39
	CF+SI	529.17±19.2bC	3287.1±81.8aA	0.16±0.01aA	13.12
	CF+DI	729.69±28.03aA	3266.5±51.3bB	0.22±0.01bAB	12.98
Rice	CK	5884.31±351.48dC	5966.7±135.6cB	0.99±0.07cAB	15.38
	CF	9801.49±699.27abA	8376.7±189.6bA	1.17±0.06aA	
	OF	10273.82±476.31aA	8891.1±111.1aA	1.16±0.04abA	0.85
	SRF	8631.85±273.50bcAB	8633.3±155.0abA	1.00±0.05bcAB	14.53
	OF+UI	7543.01±74.38Cbc	8990.0±140.1aC	0.84±0.02cB	28.21
	OF+CT	9923.72±396.77aB	8697.9±121.3aB	1.14±0.05aA	12.43
	CF+SI	7678.89±534.47ccC	7287.2±103.9bA	1.05±0.04cAB	14.98
	CF+DI	10863.92±516.34abA	8981.1±141.1aA	1.20±0.02cB	11.67

Lowercase letters indicate significant differences between treatments (*P*<0.05), and while capital letters indicate significant differences between treatments (*P*<0.01); ± show the standard errors (n = 3) of the replications.

#### Greenhouse gas emission intensity (GHGI) under different fertilization treatments

Different greenhouse gas emission intensities (GHGI) were measured over the entire year to year under the same treatments (Table 3). In the 3^rd^ crop rotation cycle, respective GHGI values were 0.16 ± 0.01 kg kg^-1^ and 0.23 ± 0.01kg kg^-1^ for OF+UI and CF for the wheat cropping season, and 0.84 ± 0.02 kg kg^-1^ (OF+UI) and 1.17 ± 0.06 kg kg^-1^ (CF)for the rice cropping season (Table 3). By comparing the fertilization treatments with the CF treatment, the percentage of GHGI in different fertilization treatments were found to be lower than that of the local traditional fertilization method. The GHGI of the CK treatment was significantly reduced by up to 15.38% for the rice cropping season, though there was no reduction detected with the wheat cropping season relative to the CF treatment; this indicated that the application of nitrogen fertilizer during the rice season led to a significant increases in GHGI, which resulted in a very significant increase in greenhouse gas emissions. Compared with CK, the GHGI of the OF treatment was significantly decreased by 17.39% for the wheat season, but there was no significant reduction with the rice season. The GHGI values of the SRF treatment were reduced by 14.53% and 26.09% over the rice and wheat seasons, respectively; this indicated that the use of controlled fertilizer could achieve significant emission reductions and yield increases with rice-wheat rotation farmland in Chaohu. The CF+SI and CF+DI treatments resulted in GHGI emission reductions of up to 14.98% and 11.6% for the rice season, respectively. Under the same treatment, GHGIs achieved a significant reduction in the wheat season.

## Discussion

Several previous studies have shown that application of nitrogen fertilizer increases the N_2_O emissions from agricultural soils [16,24,31]. N_2_O emission fluxes from rice-wheat cropping fields ranged between 0.61 μg m^-2^ h^-1^ to 1707.08 μg m^-2^ h^-1^ over the experimental period, which agreed with results from previous studies (0.6 μg m^-2^ h^-1^ to 1516.2 μg m^-2^ h^-1^) conducted in different regions [11,32,33]. In this study, a negative N_2_O emission flux was also observed in October to March, which may have been due to decreased soil temperatures. Another study reported a negative N_2_O emission flux from November to January [11]. In a terrestrial environment, there are numerous factors affecting N_2_O emissions from denitrification, nitrification, chemodenitrification, heterotrophic nitrification, codenitrification and oxidation of ammonia; these processes are directly affected by the application of nitrogen fertilizer in the soil [13,34,35].The results of this study also support this conclusion. In the same way, we analyzed the effects of nitrogen application on N_2_O emissions and emission peaks during the rice and wheat cropping seasons. In this study, nitrogen fertilizer was not used in the CK treatment, and both its seasonal and annual N_2_O accumulated emissions were significantly lower than the other fertilization treatments. Seasonal N_2_O emissions fluxes observed by Zou *et al*.[31] averaged a very low 2.26 μg m^-2^ h^-1^ with nitrogen fertilizer applied at 150 kg ha^-1^. Similarly, with reduced nitrogen fertilizer application to different agricultural fields [36–38], lower N_2_O emissions fluxes were reported. This study found that increasing the application of nitrogen fertilizer could promote N_2_O emission from soil into the atmospheric environment.

This study demonstrated that the crop rotation cycle significantly affected the emission of N_2_O in the soil, whereas reduced application of nitrogen fertilizer can decrease N_2_O emissions. Similarly, previous studies also reported that a proper crop rotation cycle can significantly reduce N_2_O emissions [13,26,33,39]. N_2_O emissions from rice and wheat were balanced during the rice-wheat cropping seasons, accounting for 55% -61% over the wheat season and 39% -44% over the rice season; this indicated that rice and wheat were the main N_2_O emission sources. Liu *et al*.[11]showed similar results for a wheat-maize crop rotation system. Over the entire experimental period, dry land and flooded paddy fields were the main sources of N_2_O emission. The results showed that N_2_O emissions could be significantly reduced up to 12.44% and 15.82% in rice and wheat compared with conventional fertilization, respectively; this could serve as the primary method for reducing N_2_O emission in the rice-wheat cropping systems in Chaohu. Our results were similar to those estimates observed by Hu *et al*.[28] in rice-wheat crop rotation cycle.

Emissions of CO_2-eq_ in the rice-wheat cropping system ranged from 341.02 ± 24.48 CO_2_-eq kg ha^-1^ to 10863.92 ± 516.34 CO_2_-eq kg ha^-1^ (Table 3), which was within the 295.65 ± 12.54 CO_2_-eq kg ha^-1^ to 9710.12 ± 474.98 CO_2_-eq kg ha^-1^ range observed in recent studies performed in the same region [23,40–42]. Consistent with previous recent studies [42–44], rice fields had greater contributions towards total CO_2-eq_ emissions than wheat fields in the rice-wheat cropping system.

Factors such as soil temperature, soil water content, rainfall and soil EC influence N_2_O emissions from agricultural soils [45,46]. Soil temperature and moisture affect the functional activity of denitrifies and nitrifiers, the production of substrates and the transport of produced N_2_O within the soil [47]. During the entire experiment, soil temperature and WFPS were considered the main factors influencing N_2_O emissions. Generally, N_2_O is emitted during soil denitrification and nitrification processes [48,49], which are highly related to soil temperature [32,49,50]; thus, soil temperature can greatly influence N_2_O emissions. Increased emissions of N_2_O as soil temperature increased from 25°C to 30°C showed that production of N_2_O was sensitive to soil temperature [6]. In this study, the average soil temperature was 15.6°C with a range of -3.1°C to 34.5°C (Fig 2A). Maximum N_2_O emissions were observed at 27.5°C, which was similar to results from recent studies [6,51]. Chang *et al*. [52] had examined the response of N_2_O and CO_2_ emissions fluxes to elevated soil temperature and showed that the rates of N_2_O and CO_2_ emissions enhanced exponentially with increases in soil temperature. Consistent with recent researches[53–55], WFPS also greatly influenced the production and emission of N_2_O from terrestrial environments. In this experiment, WFPS values ranged from 34.9% to 59.2% for both rice and wheat cultivation (Fig 2A)., which fell within the range of values (12.7 to 53.8%) observed in agricultural fields in Tennessee [38]. Different studies also reported that optimum WFPS for N_2_O emission was within the range of 48%-85% [56][57][58].

In this experimental study, the values of GHGI with different nitrogen fertilizer treatments ranged from -0.03 ± 0.01 kg CO_2_-eq ha^-1^ to 1.18 ± 0.02 kg CO_2_-eq ha^-1^ (Table 3), which was similar (0.02 ± 0.02 kg CO_2_-eq ha^-1^ to 1.15 ± 0.05 kg CO_2_-eq ha^-1^) to a previous study [44]; these were higher than values obtained from maize cropping fields in central Nebraska where the GHGI was 0.8±0.02 kg CO_2_-eq ha^-1^ [59], but lower than the 8.3 kg CO_2_-eq ha^-1^ previously estimated in China [60].

Excessive use of chemical nitrogen fertilizer application rates in rice-wheat cropping systems in China is well documented, and leads to substantial emissions of N_2_O. Future reductions of N_2_O emissions from rice-wheat cropping systems will require come critical measurements; firstly, we can reduce GHG emissions generated from nitrogen fertilizer by optimizing the application rate [61,62]. Secondly, emissions of N_2_O can also be decreased by using polymer coated fertilizers [63] and/or nitrification inhibitors [64]. In our study, among all the nitrogen fertilizer treatments, the OF+UI treatment showed maximum crop yield as well as the lowest N_2_O emissions in a rice-wheat cropping system in China. Nevertheless, with excessive use of the rice-wheat crop rotation cycle in China, there is an urgent need for proper rice-wheat cropping system specific fertilizer management optimization approaches in order to simultaneously improve crop yield and mitigate GHGs in China.

## Conclusion

In this experiment, we studied the seasonal annual N_2_O emission fluxes and crop yields under different nitrogenous fertilizer treatments (N-fertilizer) in rice-wheat cropping system from 2012–2015 in eastern China. Excessive use of N- fertilizer in rice-wheat cropping season for maximizing crop yield in China has been responsible for N_2_O emission. We also determined that different environmental factors were also involved in the emission of N_2_O. The emission fluxes of N_2_O in rice-wheat cropping season were ranged from 0.61 μg m^-2^ h^-1^ to 1707.08 μg m^-2^ h^-1^. We analyzed that N_2_O fluxes were increased by increasing the N-fertilizer application rate (0–225 kg ha^-1^). During this experiment, we also analyzed that by increasing the utilization rate of NPK fertilizers were significantly reduced the greenhouse gas emission (57.14% to 68.38%). Among all the treatments, SRF and OF+UI were found the best treatments for obtaining higher yield with less N_2_O emissions, and thus the great greenhouse gas emission reduction was also found in these treatments. The present study emphasizes that the improved management of N-fertilization significantly mitigated the emission of greenhouse gases especially, nitrous oxide form terrestrial environment to atmospheric environment and increased the crop yield.

## Supporting information

S1 Table Fertilizer application plan (kg ha^-1^) for the rice-wheat cropping system (2012–2015). (DOC)
