# Supplementary material for: Effects of fertilizer application schemes and soil environmental factors on nitrous oxide emission fluxes in a rice-wheat cropping system, east China
Source: PLoS One. 2018 Aug 14;13(8):e0202016. doi: 10.1371/journal.pone.0202016 (PMC6091932; doi:10.1371/journal.pone.0202016)
Supplement: S1 Table — (DOC) [file pone.0202016.s001.doc]

**Effects of fertilizer application schemes and soil environmental factors on nitrous oxide emission fluxes in a rice-wheat cropping system, east China**

**Awais Shakoor**^1^**, Yunlian Xu**^1^**, Qiang Wang**^1^**, Ningyi Chen**^1^**, Fei He**^1^**, Huaifeng Zuo**^1^**, Hanxun Yin**^1^, **Xiaoyuan Yan^2^, Youhua MA**^1^**^*^, Shuyun Yang**^1^**^*^**

^1^School of Resources and Environment, Anhui Agricultural University, Hefei 230036, China

^2^The Institute of Soil Science, Chinese Academy of Sciences, Nanjing, 210008, China

Corresponding Authors: YouHua Ma Email: [yhma@ahau.edu.cn](mailto:yhma@ahau.edu.cn);

ShuYun Yang Email: [yangshy@ahau.edu.cn](mailto:yangshy@ahau.edu.cn)

| wheat | T* | Total | | | Basal | | | Tillering stage | Panicle stage | |
| --- | --- | --- | --- | --- | --- | --- | --- | --- | --- | --- |
|  |  | N | P_2_O_5_ | K_2_O | N | P_2_O_5_ | K_2_O | N | N | K_2_O |
|  | CK | 0 | 0 | 0 | 0 | 0 | 0 | 0 | 0 | 0 |
|  | CF | 210 | 72 | 72 | 72 | 72 | 72 | 69 | 69 | 0 |
|  | OF | 210 | 90 | 135 | 115.5 | 90 | 94.5 | 42 | 52.5 | 40.5 |
|  | SRF | 210 | 72 | 72 | 210 | 72 | 72 | 0 | 0 | 0 |
|  | OF+UI  OF+CT  CF+SI  CF+DI | 210  210  210  210 | 90  90  72  72 | 135  135  72  72 | 115.5  115.5  72  72 | 90  90  72  72 | 94.5  94.5  72  72 | 42  42  69  69 | 52.5  52.5  69  69 | 40.5  40.5  0  0 |
| Rice | T* | Total | | | Basal | | | Tillering stage | Panicle stage | |
|  |  | N | P_2_O_5_ | K_2_O | N | P_2_O_5_ | K_2_O | N | N | K_2_O |
|  | CK | 0 | 0 | 0 | 0 | 0 | 0 | 0 | 0 | 0 |
|  | CF | 180 | 67.5 | 67.5 | 67.5 | 67.5 | 67.5 | 67.5 | 45 | 0 |
|  | OF | 225 | 67.5 | 120 | 90 | 67.5 | 84 | 90 | 45 | 36 |
|  | SRF | 180 | 67.5 | 67.5 | 180 | 67.5 | 67.5 | 0 | 0 | 0 |
|  | OF+UI  OF+CT  CF+SI  CF+DI | 225  225  180  180 | 67.5  67.5  67.5  67.5 | 120  120  67.5  67.5 | 90  90  67.5  67.5 | 67.5  67.5  67.5  67.5 | 84  84  67.5  67.5 | 90  90  67.5  67.5 | 45  45  45  45 | 36  36  0  0 |

**S1.** Fertilizer application plan (kg ha^-1^) for the rice-wheat cropping system (2012-2015).
